# Physiological reports: Standing openly for sound science

**DOI:** 10.14814/phy2.15532

**Published:** 2022-12-02

**Authors:** Josephine C. Adams

**Affiliations:** ^1^ School of Biochemistry University of Bristol Bristol UK

It is my honor and pleasure to be appointed as the next Editor‐in‐Chief of *Physiological Reports*. I thank The Physiological Society (TPS) and the American Physiological Society (APS) for the trust placed in me. I am delighted to follow the steps of the Founding Editor, Sue Wray, and the current Editor, Tom Kleyman. I thank Tom and Sue for their wise words and encouragement on approaching the transition.

*Physiological Reports* has a unique place in relation to the other journals of TPS and APS. It is a young journal that will celebrate its tenth anniversary of publication in December 2023. From its inception, it has been a fully open‐access, online journal supported by the publisher Wiley. *Physiological Reports* has a distinct mission to publish sound science and research papers that are accepted “on the basis of scientific rigor, adherence to technical and ethical standards, and evidence that the study is sufficiently well‐conceived and the data support the conclusions” (https://physoc.onlinelibrary.wiley.com/journal/2051817x). Beyond assessing the concept and scientific question addressed by the research, editors look for well‐defined experimental design, validation by replicate experiments, transparent figure presentation, and, where relevant, appropriate statistical analysis.

The emphasis on sound science makes *Physiological Reports* an intrinsically inclusive journal in terms of content. Providing the sound science criteria are met, the research advance may relate to any area of physiology and can be descriptive, correlative, confirmatory, or overlapping with prior publications, or, indeed, report negative outcomes. The authors who wish to submit novel and/or mechanistic research studies are also welcome; yet a full mechanistic explanation of the process under study is not a requirement for publication. Indeed, it is of value for authors to know that they can submit their manuscript to *Physiological Reports* for a rapid route to peer‐reviewed, final publication in an open‐access journal that is associated with esteemed professional societies and a major publisher. The publication of sound science papers is valuable in many ways. As a general principle, it is important that the outcomes of research, whether novel or not, should reach the public domain. More specifically, demonstrations of reproducibility are essential on the long path from basic discovery research to clinical translation. Reporting of negative results and the disproving of hypotheses support the advancement of a field by avoidance of future investigation of “dead ends” and help to focus research attention onto alternative hypotheses. Publication of novel findings for which a full mechanistic understanding is currently lacking helps authors to move on in their research and may assist with future funding applications.

Pursuant to these goals, *Physiological Reports* provides an efficient peer review and publication process for the authors. About 50% of manuscripts arrive by transfer from other journals of TPS and APS, *Acta Physiologica* published by the Scandinavian Physiological Society, or certain physiology‐related journals published by Wiley (Table 1). The inclusion of initial peer review reports with transferred manuscripts enables editors to make rapid decisions based on the sound science criteria and to feedback to the authors the key points in revision that should be addressed before acceptance can be considered (e.g., items of figure presentation or text changes to clarify experimental details or interpretation of data). For manuscripts that arrive by direct submission, the handling Editor will assign relevant expert reviewers from the Editorial Board or the wider field to undertake full peer review. Between January 2022 and October 2022, the mean time to first decision has been 22.8 days and the mean time from acceptance to the publication of record has been 42 days.

**TABLE 1:** Transferring supporter journals of *Physiological Reports*.

Organization	Transferring journals
American Physiological Society	*Advances in Physiology Education* *American Journal of Physiology ‐ Cell Physiology* *American Journal of Physiology ‐ Endocrinology and Metabolism* *American Journal of Physiology ‐ Gastrointestinal and Liver Physiology* *American Journal of Physiology ‐ Heart and Circulatory Physiology* *American Journal of Physiology ‐ Lung Cellular and Molecular Physiology* *American Journal of Physiology ‐ Regulatory, Integrative and Comparative Physiology* *American Journal of Physiology ‐ Renal Physiology* *Journal of Applied Physiology* *Journal of Neurophysiology* *Physiological Genomics*
The Physiological Society	*Experimental Physiology* *The Journal of Physiology*
The Scandinavian Physiological Society	*Acta Physiologica*
Wiley	*Journal of General Physiology* *Journal of Neuroendocrinology* *Journal of Pineal Research* *Journal of Sleep Research* *Psychophysiology*

My vision for *Physiological Reports* centers on two themes: to grow both the journal content and the visibility of the published articles. These goals are intrinsically linked together: by growing the breadth and depth of research topics published by the journal, it will become more visible to a wider readership and should attract new authors; by including strategies to make the published papers as visible as possible, we aim to attract in new readers and authors and encourage published authors to return. We will progress these goals through a suite of new initiatives. As an open‐access journal listed in PubMed, *Physiological Reports* is inherently inclusive to readers: articles can be discovered by any researcher or member of the public anywhere in the world who has access to the internet and a search engine. With regard to both journal content and visibility, we will work on the question of inclusivity for the authors. In the period January 2021 to date, the journal has published articles from corresponding authors in 45 countries across six continents (Figure 1). This is good news; yet, with most papers originating from North America and Europe, there is scope for *Physiological Reports* to grow further in terms of representing a truly global authorship. In addition to its role in publishing papers relevant to worldwide human health issues (e.g., cardiovascular disease), *Physiological Reports*, with its remit for sound science in basic and translational physiology, is also well placed to publish papers that reflect distinct physiology research or clinical priorities from different regions of the world. We encourage such submissions and aim to be as transparent and clear as possible about the scientific requirements for achieving publication of your paper in *Physiological Reports*. For corresponding authors based in low‐ or lower‐middle‐income countries, the open access article processing fee should not be a deterrence to submissions because the publisher Wiley is a participant in Research4Life (https://www.research4life.org) and provides fee waivers or discounts; the authors should request these when submitting their manuscript. This fee waiver process is handled entirely by the publisher and is kept separate from the scientific peer review (https://authorservices.wiley.com/open‐research/open‐access/for‐authors/waivers‐and‐discounts.html). For authors in other countries, transformative agreements, such as Wiley's Open Access accounts with funders or institutions, may be available to cover the article processing fee in whole or in part.

**FIGURE 1:**
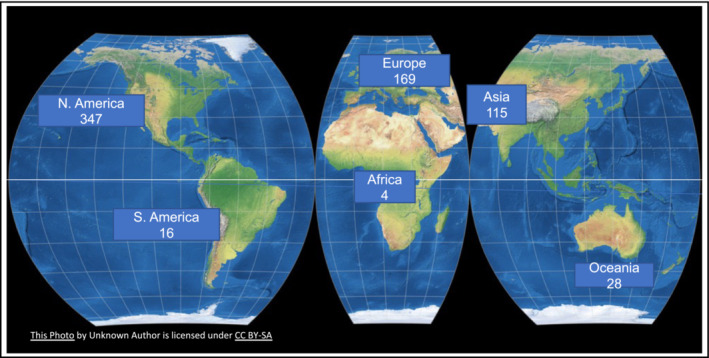
Sources of papers published in *Physiological Reports* between January 1, 2021 and October 1, 2022, according to location of corresponding authors within the geographic continents. Open source world map licensed under CC‐BY‐SA.

I am pleased to introduce a new team of Associate Editors whose collective expertise spans broad areas of physiology and who work in different regions around the world. In addition, the diversity of the Editorial Board is an important goal under the equality, diversity, and inclusion programs of APS, TPS, and Wiley. How the diversity of a Board relates to the output of publications in life sciences journals is not yet well‐understood, but a study of journals in the communications field reported that journals with more diverse Editorial Boards publish a greater diversity of research articles, based on country of origin of the research (Goyanes & Demeter, 2020). By expanding the Editorial Board to incorporate a panel of experts with increased representation with regard to geographic regions, ethnicity, sex, and other attributes of diversity, *Physiological Reports* will be in a robust position to evaluate and publish papers that reflect physiological research priorities around the world. This process has started and will continue in 2023.

Citation is a key metric of a paper's interest to its field and papers in *Physiological Reports* can certainly achieve rapid, extensive citation. For example, the top‐cited research paper of the last 2 years (Lemes et al., 2021) achieved 19 citations at the time of writing and is in the top 10% of papers tracked by altmetrics, while the top‐cited Review (Østergaard, 2021) has achieved 78 citations and is in the top 5% of papers with altmetrics. Nowadays, many routes outside PubMed are used by authors to track and share new publications in their field; for example, Google scholar or social media are increasingly used. Although there are conflicting reports on whether social media attention can enhance citation rates (some studies report a weak positive correlation, e.g., Costas et al., 2014, Barakat et al., 2018, whereas others found no correlation, e.g., Noah et al., 2020), there is evidence that articles which receive high views/downloads soon after publication are candidates for strong citation (Bommann & Haunschild, 2018). In recognition of the plurality of routes by which readers identify articles of interest and the importance of visibility upon publication, we plan to expand activities to promote journal content in the first months after publication. As examples, we will increase the frequency of topical Calls for Papers and (in addition to promoting single papers in social media or by press release) will make more systematic use of collections of related papers, as they are built up on publication, through a Call or by matched keywords. With regard to longer‐term impact, in recent years *Physiological Reports* has offered poster prizes for early‐career researchers at key international physiology conferences. We aim to maintain this tradition as well as setting up a “Paper of the Year” award to showcase the quality and significance of the very best research published in *Physiological Reports* each year.

While research articles form the majority of the papers published, *Physiological Reports* also publishes Case reports, Short Invited Reviews, and occasionally, Methods articles. Appropriate methodology is an essential component of sound science and I plan to develop the publication of Methods articles by establishing this as a separate category of paper with clear format guidelines. Once again inter‐twining the themes of journal content and journal/article visibility, the Short Review format is ideal for succinct updates on current physiological topics. We will also be looking to align suites of Reviews with current initiatives and priorities of APS and TPS. For example, TPS has an ongoing strategic goal to drive forward research and knowledge related to “Physiology and Climate Change.” Aspects of the physiology of all living organisms are susceptible to impact by climate change, and atmospheric pollution, extreme heat, and drought are already affecting the health of humans and animals. Integration between traditional disciplines in physiology, between physiologists in different countries, and between physiologists and other fields are needed for research to progress rapidly. I look forward to ensuring that *Physiological Reports* plays its best part in this initiative. Ongoing policy and advocacy initiatives at APS on “Animal Research” and “Reproducibility in Research” are also very relevant to the mission and content of *Physiological Reports*.

The training and education missions of APS and TPS are essential to the continued vitality and rigor of research and publications in the field of physiology. The “sound science” mission of *Physiological Reports* is very apt to the training of new reviewers and Editors through hands‐on experience. I have marked this by inclusion of early‐career independent researchers among the first new appointments to the Editorial Board and, with the support of the Associate Editors, aim to maintain a rolling annual program of early‐career researcher new appointments in the next years. The senior Editors' team will also seek opportunities to engage with the APS' “Career Gateway” and “Centre for Physiology Education” and the TPS' “Professional Development” portfolios and to champion the strong representation of reviewer and editor training materials within these online resources. We will reach out to early‐career Society Council members to identify the best ways for this career‐stage cohort to engage with *Physiological Reports*.

Since the founding of *Physiological Reports* in 2013, the life sciences publishing “ecosystem” has changed dramatically and continues to change. Funding body mandates for open access publication have moved from isolated examples to the norm. Pre‐print servers now offer authors an instantaneous way to publicize manuscripts online without formal peer review. A new manuscript handling process has been started at the journal *eLife* such that all manuscripts selected for peer review will be presented online along with peer review reports as “reviewed preprints,” removing the final step of acceptance and publication (Eisen et al., 2022). Nevertheless, for many researchers, funding bodies and students, the final paper of record, that has been independently peer‐reviewed, publisher‐checked for figure presentation quality and text originality, and copy‐edited, remains an essential resource. Within this swift flow of change, *Physiological Reports* stands stable as an established, fully Open access, peer‐reviewed journal that draws strength from the collaboration of two major, non‐for‐profit, professional Societies. It offers the authors and readers a trusted forum for publication and distribution of knowledge and scholarship in physiology. As 2023 gets underway, I look forward to starting the new initiatives and working with the Associate Editors and Board, the Societies, and the publisher to ensure that *Physiological Reports* provides the best possible service and resources to the authors and readers. Please submit your papers at https://physrep.msubmit.net. If you have ideas for Reviews, Methods topics, or Calls for Papers, please contact myself as Editor‐in‐Chief at jo.adams@bristol.ac.uk. General enquiries for submissions can be made through the Journal's dedicated email address physiologicalreports@wiley.com .

## CONFLICT OF INTEREST

No conflicts of interest are declared.
